# Developmental Deficiency of Oligodendrocytes and Myelination in the Central Nervous System of the Fetal Alcohol Syndrome Mouse Model

**DOI:** 10.1002/cns.71157

**Published:** 2026-09-11

**Authors:** Xiaoling Liang, Rulan Yi, Zhidan Ke, Yi Zhang, Liang Zhou

**Affiliations:** ^1^ Key Laboratory of Anesthesia and Organ Protection of the Ministry of Education (In Cultivation) Zunyi Medical University Zunyi China; ^2^ People's Hospital of Yilong County Nanchong China; ^3^ Department of Anesthesiology The Second Affiliated Hospital of Zunyi Medical University Zunyi China

**Keywords:** fetal alcohol syndrome, myelination, oligodendrocytes, prenatal and lactation alcohol exposure, radial glia cells

## Abstract

**Background:**

Fetal alcohol spectrum disorder (FASD) is a severe condition characterized by physical and neurodevelopmental abnormalities in the fetus resulting from prenatal alcohol exposure (PAE) or prenatal and lactation alcohol exposure (PLAE). Behavioral abnormalities associated with white matter damage have been extensively investigated in FASD patients and animal models, which suggested the involvement of myelination in FASD pathology. However, the impact of FASD on oligodendrocytes (OLs) and myelination within the central nervous system (CNS) and the underlying mechanisms are little discovered. This study aimed to reveal the effect and role of PLAE on CNS myelination in a FASD mouse model.

**Methods:**

We set up a PLAE approach to generate the FASD mouse model to explore the effects of FASD on CNS myelination. Open‐field test, elevated plus maze, and tail suspension test were used to evaluate possible behavior deficiency of the FASD mice. Myelination in the cortex and hippocampus of the FASD mice was detected at postnatal days 14, 21, and 90 (P14, P21, and P 90). Then we counted the number of oligodendrocyte precursor cells (OPCs) and mature oligodendrocytes (mOLs) to reveal the cell fates of oligodendrocyte lineage cells in the FASD mice. Next, we set up BLBP+/GFAP+ double‐staining to monitor the possible dysfunction of radial glial cells, which may contribute to the myelination deficiency in FASD mice. Finally, an FDA‐approved pro‐myelination drug, clemastine fumarate (CF), was used to validate the possible advantages of myelin restoration and behavior defects in the FASD mice.

**Results:**

Impaired myelination was observed in the cortex and hippocampus of the male FASD offspring, associated with hyperactivity‐like behaviors, whereas the female FASD mice exhibited delayed myelin formation and normal behaviors, indicating a gender‐specific manner of FASD pathology. Furthermore, the different myelination deficiency between male and female FASD mice is due to the fact that only the male FASD mice exhibited a failure of oligodendrocyte differentiation. Additionally, FASD exerts an impact on the quantity and differentiation of radial glia cells, which may contribute to the myelination defect. Finally, CF‐treated male FASD mice showed myelin regeneration, leading to partial alleviation of behavioral abnormality in the male FASD mice.

**Conclusion:**

Our data reveal a gender‐related deficiency of CNS myelination in the FASD mouse model, suggesting a potential therapy target for FASD.

## Graphical abstract

Impaired myelination was observed in the central nervous system (CNS) of the male fetal alcohol spectrum disorder (FASD) offspring associated with hyperactivity‐like behaviors, whereas the female FASD mice exhibit delayed myelin formation and normal behaviors, indicating a gender‐specific manner of FASD pathology.

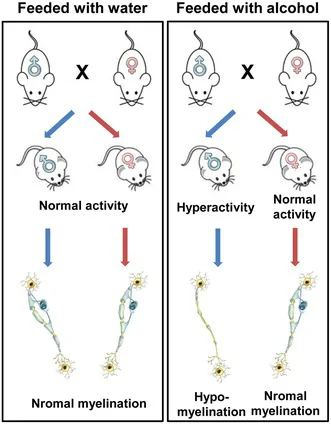

## 1 Introduction

Prenatal alcohol exposure (PAE) or prenatal and lactation alcohol exposure (PLAE) exerts a profound impact on the developing fetus and can lead to fetal alcohol spectrum disorder (FASD). Numerous studies have demonstrated that the characteristics of FASD encompass atypical physical development alongside central nervous system (CNS) dysfunction, including cognitive impairment [1, 2, 3]. Even minimal PAE can exert discernible impacts on the structural integrity of children's brains [4]. Alcohol exposure exerts enduring detrimental effects on various neuronal populations in the developing brain, leading to impairments in higher‐order cognitive functions such as learning and memory, emotional regulation, and motor control [5, 6, 7]. In addition to neurons, alcohol exposure also induces damage to glial cells, including astrocytes and microglia, which have been shown to participate in the regulation of cognitive functions and neuroinflammation in the CNS [8, 9, 10, 11, 12].

Furthermore, neuroimaging studies revealed abnormal connectivity of white matter in FASD patients or animal models, which in turn manifests as reduced attention, behavioral disorders, and hyperactivity disorders [13, 14, 15, 16]. In the CNS, oligodendrocytes (OLs)‐formed myelin sheath, as the major component of white matter, is essential for neuron integrity and functions. Growing evidence has shown the critical roles of OLs and myelin in neuronal activity and cognitive functions in health and disease, including motor learning, memory consolidation, and autism [17, 18, 19, 20], indicating myelin as a potential intervention target for multiple brain dysfunctions. In the FASD mouse model, white matter damage was shown to be accompanied by oligodendrocyte loss [21, 22], suggesting the potential deficiency of myelination in FASD. However, the precise impact of PAE or PLAE on oligodendrocytes and myelination remains uncertain.

The radial glial cells (RGCs) in the embryo possess the ability to give rise to both neurons and glial cells, including OPCs [23]. PAE results in a reduction in the RGC population and exerts an impact on neurogenesis and astrocyte generation [24]. On the other hand, PAE was also found to induce alterations in glial development, thereby expediting the transition from RGCs to astrocytes [25, 26]. Therefore, we postulate that a reduction of the RGC population or alteration of RGC differentiation may constitute one of the mechanisms underlying oligodendrocyte damage and myelination disorder associated with FASD.

Here, we set up a FASD mouse model to demonstrate that PLAE impairs myelination in the CNS, which is associated with hyperactivity‐related behaviors in the male offspring. Furthermore, hypomyelination in FASD mice is attributed to the inhibition of oligodendrocyte precursor (OPC) proliferation and differentiation. Additionally, our findings demonstrate that FASD exerts an impact on the quantity and differentiation of RGCs, consequently influencing oligodendrocytes' development and myelination. Finally, an FDA‐approved pro‐myelination drug, clemastine fumarate (CF) treatment, showed myelin regeneration, leading to partial alleviation of behavioral abnormality in the male FASD mice. Together, our data reveal the deficiency of oligodendrocyte development and myelination in the CNS of the FASD mouse model and the possible underlying mechanism.

## 2 Materials and Methods

### 2.1 Animals

C57BL/6J mice were used for all experiments and purchased from Changsha Tianqin Technology Co. Ltd. (Changsha, China). Mice were raised in the Experimental Animal Center of Zunyi Medical University and kept under standard conditions of 21°C ± 1°C and 55% ± 10% relative humidity, with a controlled 12‐h light/dark cycle and free access to water and food.

To generate the FASD model, one male and two female C57BL/6J mice were randomly selected and put in the same cage for mating. The male was moved out until the pregnancy of the females was confirmed. The FASD groups (FASD) were supplied with a 5% alcohol solution as the only water source. The control groups (Ctrl) were supplied with standard tap water. A 5% alcohol solution was prepared by mixing 5% anhydrous ethanol with distilled water. After birth, the FASD groups were continued to be fed with a 5% alcohol solution until the young mice were weaned, which is around 21 days after birth, while the control group received regular drinking water. The age‐matched offspring were randomly picked for the following tests.

For CF treatment, the weaned male FASD mice were randomly divided into two groups; daily 10 mg/kg CF or an equal volume of saline was applied by gavage to the mice for 4 weeks.

### 2.2 Antibodies and Reagents

Antibodies against Olig2 (AB9610, RRID:AB_570666; MABN50, RRID:AB_10807410), CC‐1 (OP80, RRID:AB_2057371), MBP (SMI99, RRID:AB_2140491) and MAG (MAB1567, RRID:AB_11214010) were from Millipore (United States). Antibodies against BLBP (OB‐PGP011, RRID:AB_2938945), GFAP (OB‐MMS036, RRID:AB_2,938,930) and PDGFR‐α (OB‐PRB011, RRID:AB_2938683) were from Oasis Biofarm (China). Ptchd1 antibody (ab109407, RRID:AB_10860521) was from Abcam (United Kingdom). Antibodies against Pax6 (12323–1‐AP, RRID:AB_2159695), PSD95 (20665–1‐AP, RRID:AB_2687961), and Synaptophysin (SYP, 67864–1‐Ig, RRID:AB_2918622) were from Proteintech (China). Antibody against c‐Fos (226,008, RRID:AB_2891278) was purchased from Synaptic Systems (Germany). NF antibody (SMI‐31, 801,602, RRID:AB_2715851) was purchased from BioLegend (United States). β‐actin antibody (AC026, RRID:AB_2768234) was purchased from ABclonal (China). HRP‐conjugated secondary antibodies (#31460, RRID:AB_228341; #31430, RRID:AB_228307) were from Thermo Fisher Scientific (United States). Alexa Fluor‐conjugated secondary antibodies (A‐11008, RRID:AB_143165; A‐11012, RRID:AB_2534079; A‐11001, RRID:AB_ 2,534,069; A‐11005, RRID:AB_2534073; A‐11073, RRID:AB_2534117) were from Invitrogen (United States). EdU staining proliferation kit (iFluor 647, ab222421) was from Abcam. Proteinase inhibitor (P8340) was from Sigma‐Aldrich (United States). Clemastine fumarate (S1847) was from SelleckChem.

### 2.3 Behavior Tests

At postnatal day 90 (P90), mobility and mood‐related behaviors of the offspring from the FASD and the control groups were evaluated by the following tests:

Open‐field test (OFT). Mice were placed in the center of a rectangular gray plastic box (H24.5 x L35 x L55 cm) and recorded for 10 min. Total distance and time spent in the center of the OF were recorded using AnyMaze tracking software (Stoelting Co). The OF box was cleaned with 75% ethanol between each trial.

Elevated plus maze (EPM). The maze is a gray plus‐shaped apparatus, with two open arms (30 cm × 5 cm) and two closed arms (30 cm × 5 cm with 15 cm high walls) that connect with a central platform (5 cm × 5 cm) and is located 40 cm above the floor. Each mouse was placed on the center platform facing an open arm and allowed to move freely in the maze for 5 min. The time spent and the entries into the open arms were automatically measured using AnyMaze tracking software. The maze was wiped clean with 75% ethanol between each trial.

Tail suspension test (TST). The mice were suspended 50 cm above the floor by their tails. The tail of each mouse was securely fastened to a hook positioned 1 cm from its tip. The duration of immobility was measured during a 6‐min testing period. The state of immobility was defined as the absence of all movement except for whisker motion and respiration.

### 2.4 Immunofluorescence

Coronal sections (20 μm) were placed in a blocking solution (PBS with 1% BSA, 0.3% Triton, 10% goat serum) for 2 h at room temperature (RT), then incubated with primary antibodies overnight at 4°C and secondary antibodies for 2 h at RT. The dilutions of primary antibodies were 1:1000 for MBP and GFAP; 1:500 for Olig2 and c‐Fos; and 1:100 for NF, Pax6, CC1, PDGFR‐α, and BLBP. The secondary antibodies were diluted at 1:1000. The sections were mounted with DAPI Fluoromount‐G (Southern Biotech). An inverted laser scanning confocal microscope (FV1000, Olympus) was used for fluorescence imaging. ImageJ 1.42q (NIH) was used for image analysis.

For EdU labeling, the control or FASD mice were intraperitoneally injected with EdU (50 mg/kg) for three consecutive days prior to perfusion fixation. Then an EdU staining proliferation kit (iFluor 647, ab222421) was used to stain EdU‐labeled samples following the manufacturer's instructions.

### 2.5 Western Blotting

Proteins collected from cortex (CTX) or hippocampus (HIP) were dissolved in PBS containing 2% SDS with protease inhibitor. Protein concentration was determined by BCA assay. Equal quantities of protein were loaded onto SDS‐PAGE, transferred to a PVDF membrane (Immobilon‐P, Millipore), immunoblotted with antibodies, and visualized with an ECL kit (Pierce Biotechnology). Primary antibody dilutions were 1:1000 for PTCHD1, MAG, and BLBP and 1:10,000 for MBP, PSD95, SYP, GFAP, Pax6, and β‐actin. Digital signals were scanned by ChemiDocTM Imaging System (Bio‐Rad) and quantified using ImageJ. The immunoreactivity of β‐actin was set as the loading control, and all data were normalized to the corresponding control.

### 2.6 OPC Culture

OPCs from SD rats (P0) were cultured according to the manufacturer's standard protocol. In brief, OPCs were collected from glial cultures by shaking for 1 h at 200 rpm, incubating in fresh medium for 4 h, and shaking at 250 rpm at 37°C for 16 h. Collected OPCs were replated onto poly‐D‐lysine‐coated plates and grown in Neurobasal supplemented with 2% B27. PDGF‐AA (10 ng/ml) was added to keep OPCs undifferentiated, or triiodothyronine (T3, 40 ng/mL) was added for 3 days to allow differentiation. For alcohol treatment of OPCs, 30 mM EoTH was added with T3 for 3 days.

### 2.7 Statistical Analysis

GraphPad Prism 9.0 was used for statistical analyses. Data were presented as the mean ± SEM. Unpaired two‐sided Student's *t*‐test was performed for statistical comparison between two groups. One‐way ANOVA followed by Tukey's post hoc test was used for multiple group comparisons. The significance level accepted in all cases was set at *p* < 0.05. ‘n’ refers to the number of animals we tested in the corresponding experiments.

## 3 Results

### 3.1 Generation of the FASD Mouse Model

**FIGURE 1:**
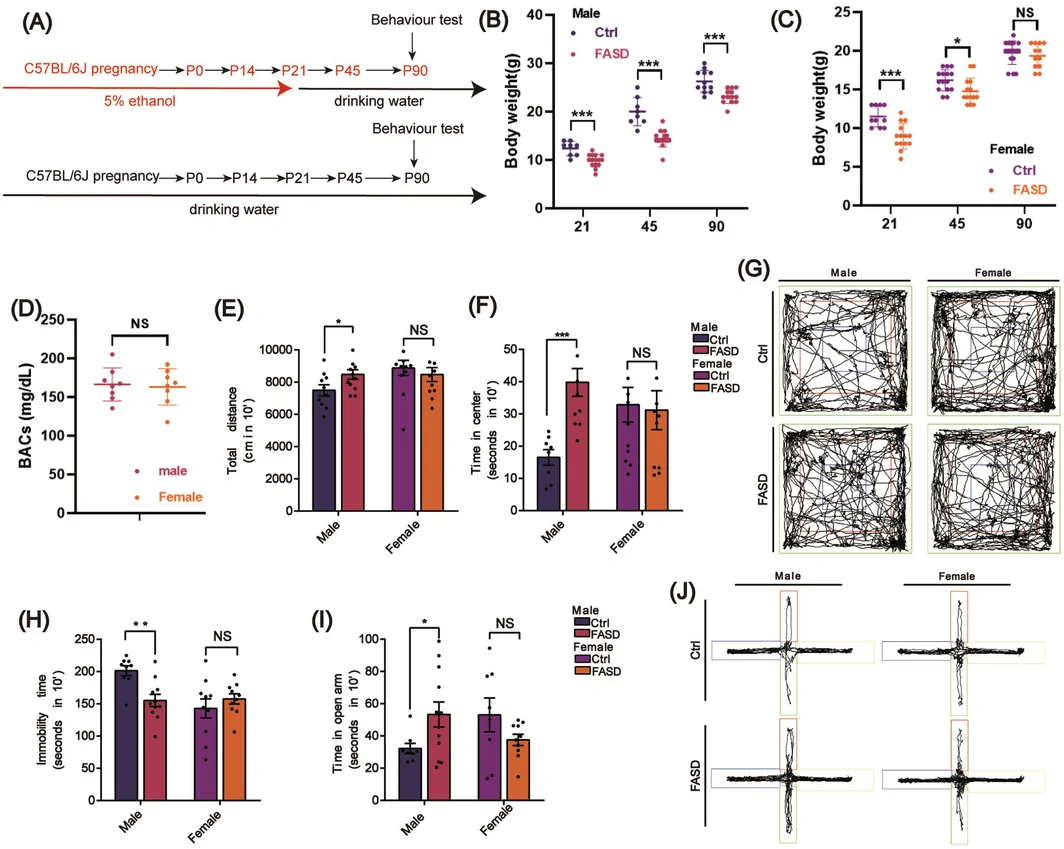
Male FASD mice exhibit hyperactivity‐related behaviors. (A) Experimental design for generating the FASD mouse model. (B) The body weight of male Ctrl and FASD mice measured at P21, P45, and P90, respectively. (C) The body weight of female Ctrl and FASD mice measured at P21, P45, and P90, respectively. (D) Blood alcohol concentrations (BACs) in male and female FASD mice at P21 (*n* = 8/group). (E) Total distance traveled in the open field (OF) of Ctrl and FASD mice (Male: Ctrl: *N* = 10; FASD: *N* = 12; Female: Ctrl: *N* = 12; FASD: *N* = 11). (F) Time spent in the center of the OFT of Ctrl and FASD mice (Male: Ctrl: *N* = 8; FASD: *N* = 12; Female: Ctrl: *N* = 8; FASD: *N* = 10). (G) Diagram of the OFT for Ctrl and FASD mice. (H) Immobility time in the TST of Ctrl and FASD mice (Male: Ctrl: *N* = 9; FASD: *N* = 11; Female: Ctrl: *N* = 10; FASD: *N* = 10). (I) Time spent in the open arms of the EPM of Ctrl and FASD mice (Male: Ctrl: *N* = 8; FASD: *N* = 12; Female: Ctrl: *N* = 8; FASD: *N* = 10). (J) Diagram of the EPM for Ctrl and FASD mice. Unpaired Student's *t*‐tests: (B–F, H, I). *p < 0.05, **p < 0.01, ***p < 0.001.

We set up a PLAE approach to generate the FASD mouse model to explore the effects of FASD on CNS myelination (Figure 1A). From P21 to P90, the male FASD mice exhibited significantly lower body weight compared to the control group (Figure 1B), while the weight of female FASD mice was lower than the control mice from P21 to P45 and recovered to be comparable to the control mice till P90 (Figure 1C). However, comparable blood alcohol concentrations (BACs) were detected in both male and female offspring at P21 (Figure 1D), which indicated that the alcohol exposure of male and female offspring is comparable. At P90, OFT, TST, and EPM were performed to characterize the FASD offspring. In the OFT, the male FASD mice exhibited an increase in total traveling distance and spent more time in the center (Figure 1E–G). Meanwhile, the male FASD mice also showed less immobility time in the TST (Figure 1H) and more time spent in the open arms in the EPM (Figure 1I,J). These data suggested hyperactivity‐like behaviors in the male FASD mice, which is partly consistent with the features of idiopathic attention‐deficit hyperactivity disorder (ADHD) after PAE [14, 15, 16]. On the contrary, the female FASD mice showed comparable behaviors to the control mice (Figure 1E–J). Together, PLAE induced hyperactivity‐like behaviors in the male FASD offspring, indicating a gender‐specific manner of FASD pathology.

### 3.2 Hypomyelination in the Male FASD Mice and Delayed Myelin Formation in the Female FASD Mice

**FIGURE 2:**
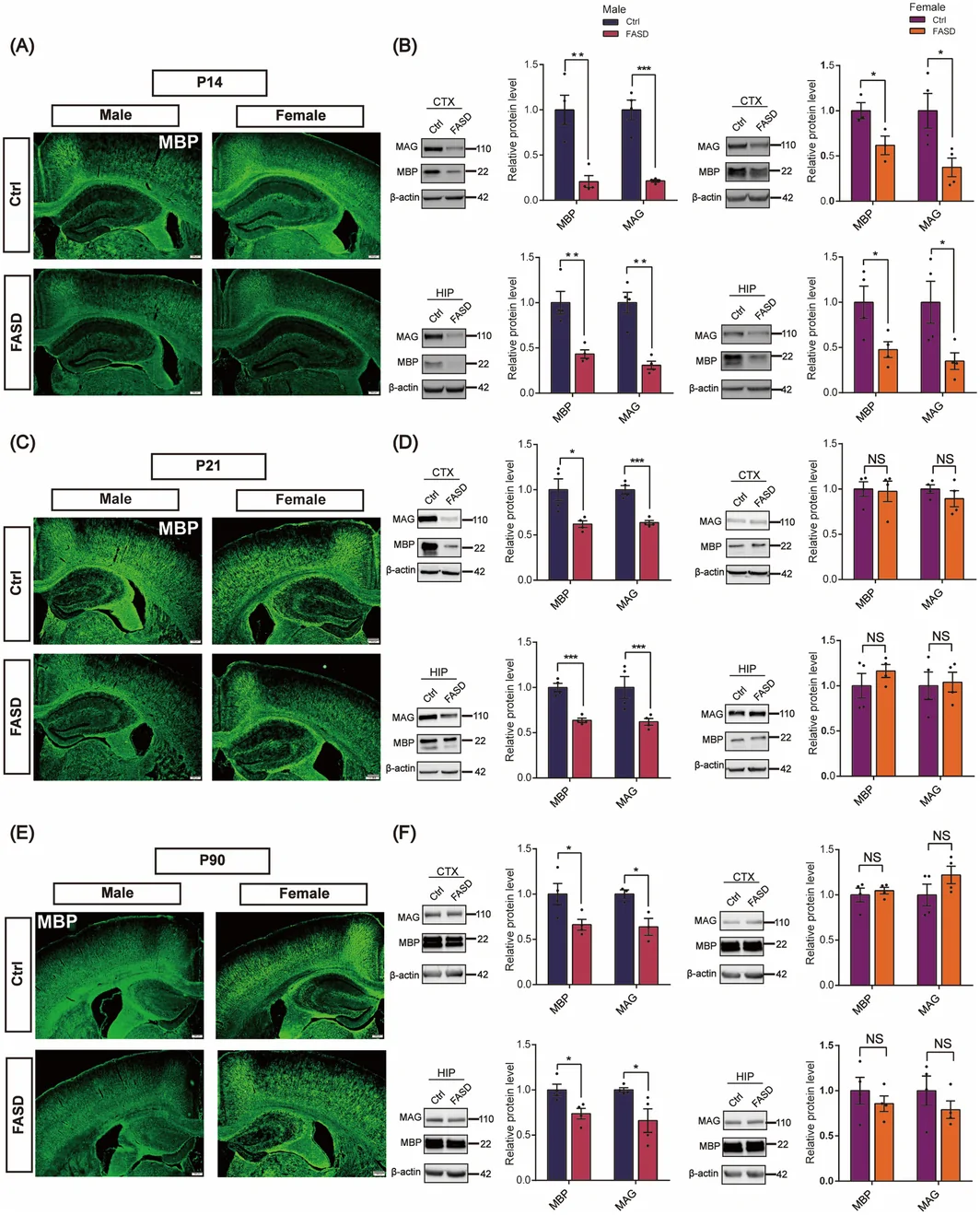
Hypomyelination in male FASD mice and delayed myelination in female FASD mice. (A) MBP staining showing myelin tracts in Ctrl and FASD mice at P14 (scale bar, 200 μm). (B) Western blot and quantification of MAG and MBP expression in the cortex (CTX) and hippocampus (HIP) of Ctrl and FASD mice at P14 (*n* = 4/group). (C) MBP staining showing myelin tracts in Ctrl and FASD mice at P21 (scale bar, 200 μm). (D) Western blot and quantification of MAG and MBP expression in the CTX and HIP of Ctrl and FASD mice at P21 (*n* = 4/group). (E) MBP staining showing myelin tracts in Ctrl and FASD mice at P90 (scale bar, 200 μm). (F) Western blot and quantification of MAG and MBP expression in the CTX and HIP of Ctrl and FASD mice at P90 (*n* = 4/group). Unpaired Student's *t*‐tests: (B, D, F). *p < 0.05, **p < 0.01, ***p < 0.001.

Neuroimaging evidence demonstrated a reduction in both gray and white matter volume in FASD patients [14]. Furthermore, an increase in alcohol consumption is associated with a reduction in residual brain volume [27]. These findings indicated a possible defect in oligodendrocyte development or myelination in FASD. So we tested myelination in the FASD mice. Different characteristics of myelination in the male and female FASD mice were revealed. MBP staining showed a weaker signal and an expanded unmyelinated area (Figure 2A,C,E; S1A–C), and western blotting revealed decreased MBP and MAG protein levels in the cortex (CTX) and hippocampus (HIP) of the male FASD mice (Figure 2B,D,F) at P14, P21, and P90, which indicated a sustained myelination defect in the male FASD mice. Meanwhile, MBP staining and western blotting showed hypomyelination in the female FASD mice only at P14 (Figure 2A,B; S1A), while the myelin density was restored to be comparable with the control mice at P21 and P90 (Figure 2C–F; S1B,C). These results suggested delayed myelin formation in the female FASD mice. Furthermore, we used MBP/NF double staining to observe the myelin ultrastructure in the CTX of male and female FASD mice at P21 (Figure S2D,E) [28]. Compared with the control mice, the male FASD mice showed significant myelin loss and axon fragmentation in the CTX, while the female FASD mice showed roughly complete myelin structure but also some axon fragmentation in the CTX. Additionally, some of the MBP‐labeled myelin was not associated with axons and was present as globules, suggesting a degree of demyelination in both genders of FASD mice. Taken together, our findings demonstrate a gender‐dependent manner of FASD in CNS myelination.

### 3.3 OPC Proliferation and Differentiation Are Blunted in the Male FASD Mice

**FIGURE 3:**
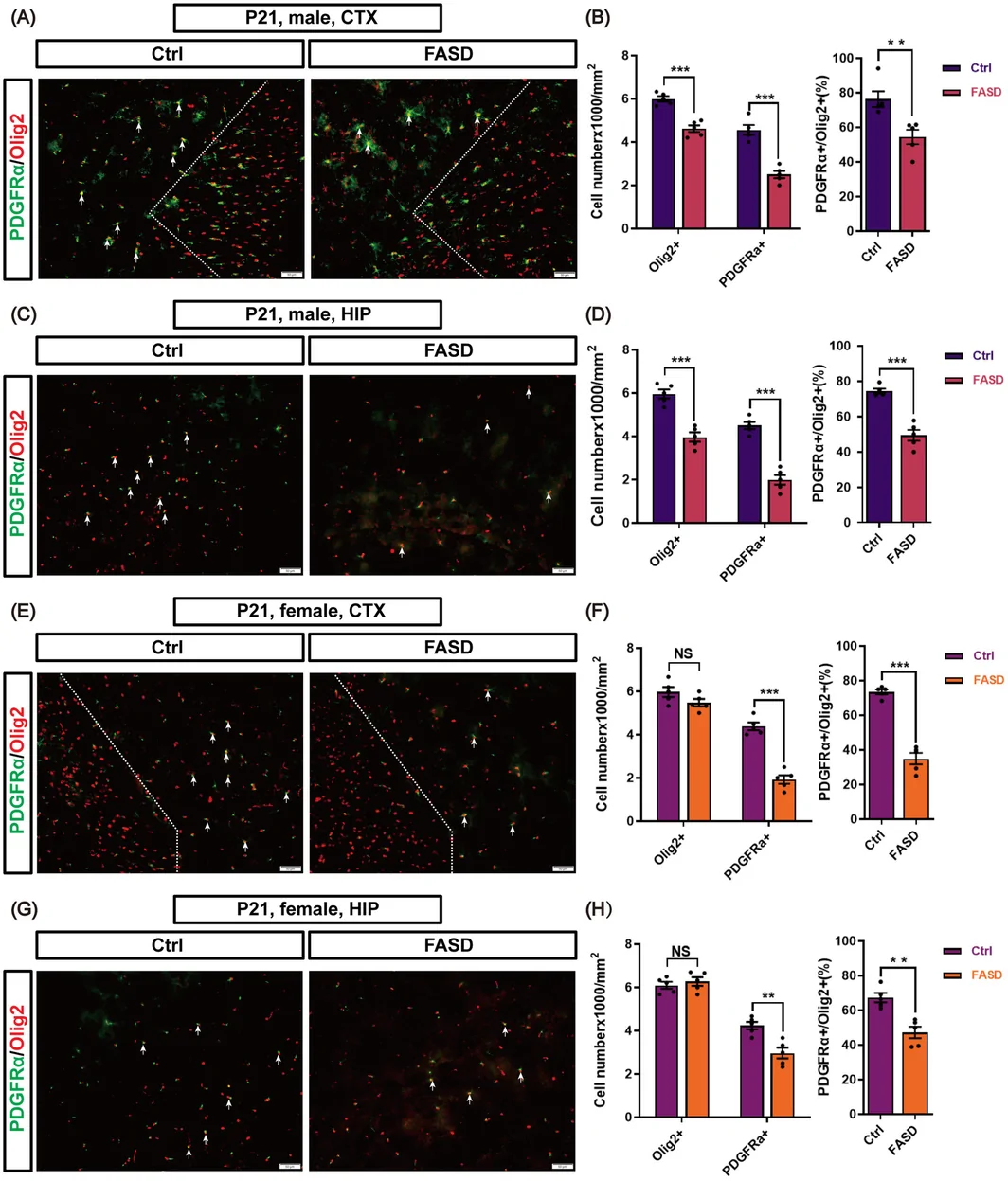
OPC proliferation is impaired in both male and female FASD mice. (A) Co‐staining of Olig2/PDGFRα showing total OLs (Olig2+) and OPCs (PDGFRα+/Olig2+) in the CTX of male Ctrl and FASD mice at P21 (scale bar, 50 μm). (B) Quantification of Olig2+ and PDGFRα+/Olig2+ cell numbers in the CTX of male Ctrl and FASD mice (*n* = 5/group). (C) Co‐staining of Olig2/PDGFRα showing total OLs (Olig2+) and OPCs (PDGFRα+/Olig2+) in the HIP of male Ctrl and FASD mice at P21 (scale bar, 50 μm). (D) Quantification of Olig2+ and PDGFRα+/Olig2+ cell numbers in the HIP of male Ctrl and FASD mice (*n* = 5/group). (E) Co‐staining of Olig2/PDGFRα showing total OLs (Olig2+) and OPCs (PDGFRα+/Olig2+) in the CTX of female Ctrl and FASD mice at P21 (scale bar, 50 μm). (F) Quantification of Olig2+ and PDGFRα+/Olig2+ cell numbers in the CTX of female Ctrl and FASD mice (*n* = 5/group). (G) Co‐staining of Olig2/PDGFRα showing total OLs (Olig2+) and OPCs (PDGFRα+/Olig2+) in the HIP of female Ctrl and FASD mice at P21 (scale bar, 50 μm). (H) Quantification of Olig2+ and PDGFRα+/Olig2+ cell numbers in the HIP of female Ctrl and FASD mice (*n* = 5/group). Unpaired Student's *t*‐tests: (B, D, F, H). **p < 0.01, ***p < 0.001.

We next analyzed the cell fates of OL lineage cells in the FASD mice at the end of the time point of early myelination in the CNS (P21). The number of Olig2‐labeled total OLs and PDGFRα‐labeled OPCs was decreased in the CTX and HIP of the male FASD mice compared to the control mice (Figure 3A–D), while the OPC number was decreased in the CTX and HIP of the female FASD mice (Figure 3E–H), demonstrating the inhibition of OPC proliferation in the FASD mice.

**FIGURE 4:**
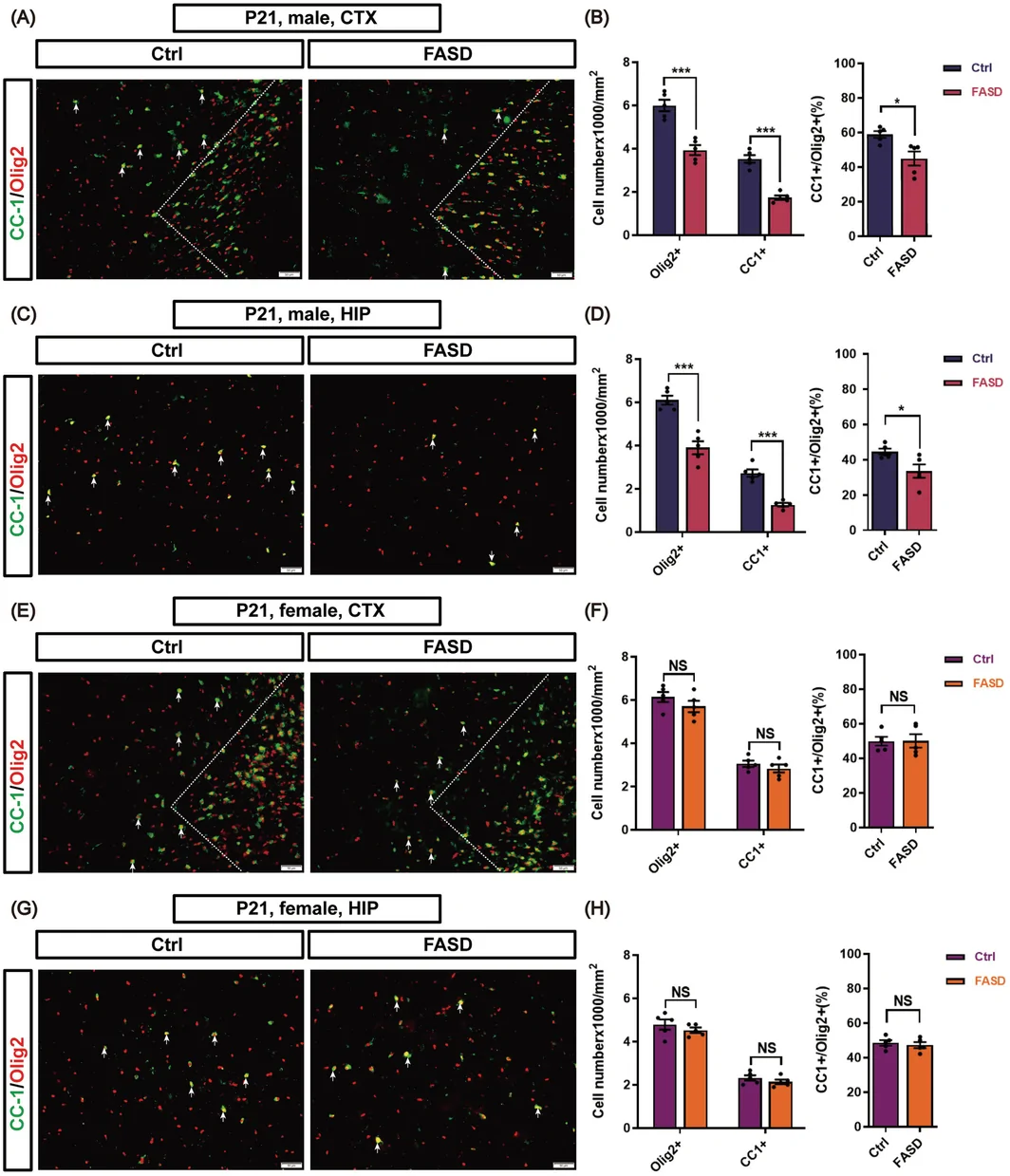
OPC differentiation is impaired in the male FASD mice. (A) Olig2/CC‐1 double‐straining showing total (Olig2+) and mature (CC‐1+/Olig2+) OLs in the CTX of male Ctrl and FASD mice at P21 (scale bar, 50 μm). (B) Quantification of Olig2+ and CC‐1+/Olig2+ cell numbers in the CTX of male Ctrl and FASD mice (*n* = 5/group). (C) Olig2/CC‐1 double‐straining showing total (Olig2+) and mature (CC‐1+/Olig2+) OLs in the HIP of male Ctrl and FASD mice at P21 (scale bar, 50 μm). (D) Quantification of Olig2+ and CC‐1+/Olig2+ cell numbers in the HIP of male Ctrl and FASD mice (*n* = 5/group). (E) Olig2/CC‐1 double‐straining showing total (Olig2+) and mature (CC‐1+/Olig2+) OLs in the CTX of female Ctrl and FASD mice at P21 (scale bar, 50 μm). (F) Quantification of Olig2+ and CC‐1+/Olig2+ cell numbers in the CTX of female Ctrl and FASD mice (*n* = 5/group). (G) Olig2/CC‐1 double‐straining showing total (Olig2+) and mature (CC‐1+/Olig2+) OLs in the HIP of female Ctrl and FASD mice at P21 (scale bar, 50 μm). (H) Quantification of Olig2+ and CC‐1+/Olig2+ cell numbers in the HIP of female Ctrl and FASD mice (*n* = 5/group). Unpaired Student's *t*‐tests: (B, D, F, H). *p < 0.05, ***p < 0.001.

On the contrary, CC‐1‐labeled mature OLs were found to be reduced only in the male FASD mice (Figure 4A–D), but not in the female (Figure 4E–H), which indicated the normal ability of OPCs to differentiate into mature OLs in the female FASD mice.

To confirm the different fates of OL lineage cells between the male and female FASD mice, we next checked the number of OPCs and mature OLs in the CTX of male and female FASD mice at P14, respectively. Olig2‐labeled OL lineage cells were found to be reduced in both male and female FASD mice, while the decreased ratio of CC‐1‐labeled mature OLs in OL lineage cells was only observed in the male FASD mice (Figure S2A,B,E,F). However, the reduction in the number of OPCs was found in both genders of FASD mice (Figure S2C,D,G,H), which is consistent with the inhibition of OPC proliferation in the FASD mice at P21 (Figure 3). Moreover, we also found abnormal morphology of OPCs in the CTX of FASD mice. OPCs in the CTX of both male and female mice exhibited shrunken areas and simplified branches, which were observed to be more severe in the male FASD mice (Figure S2C,G), indicating the different effects of PAE on OPC physiology between male and female offspring [29].

Lastly, the EdU assay was used to validate the ability of OPC proliferation in the FASD mice at P21. EdU‐labeled PDGFRα+ OPCs were significantly reduced in the CTX of the male FASD mice (Figure S3A,B), demonstrating the blunted proliferative ability in the male FASD mice. On the contrary, Edu‐labeled OPCs were comparable in the CTX between female FASD and control mice at P21 (Figure S3C,D), indicating that the loss of OPCs in the female FASD mice may occur in the early stage of postnatal development. In addition, OPCs cultured with alcohol confirmed that OPC differentiation was blocked by alcohol exposure (Figure S3E,F). These data suggested that the myelination deficiency in the male FASD mice may be due to the inhibition of both OPC proliferation and differentiation, while the decreased OPC number leads to delayed myelin formation in the female FASD mice.

### 3.4 RGC Dysfunction Contributes to the Myelination Deficiency in FASD Mice

**FIGURE 5:**
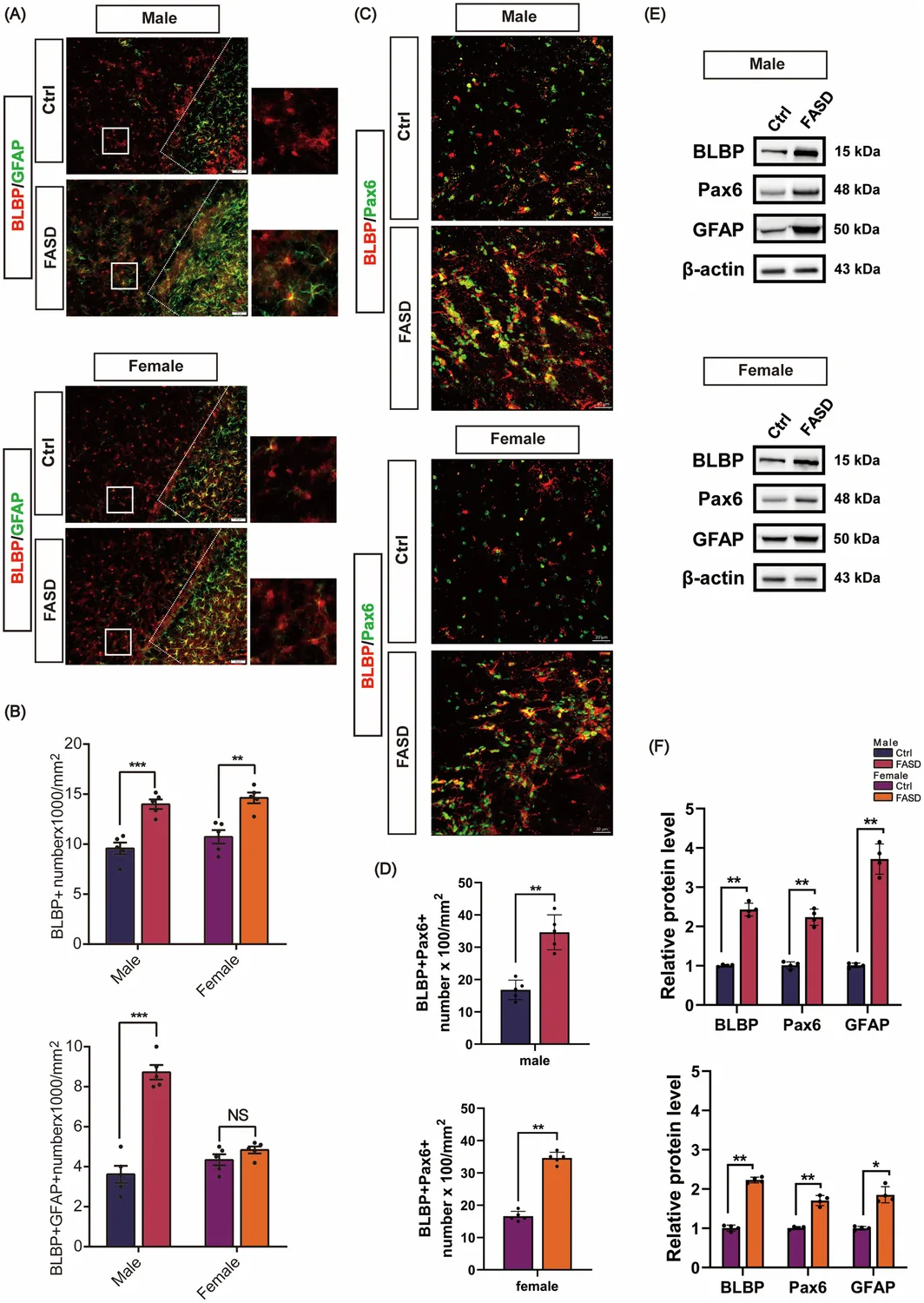
RGC dysfunction contributes to the myelination deficiency in FASD mice. (A) Co‐staining of BLBP and GFAP in the CTX of Ctrl and FASD mice at P21. Higher magnifications show the cells stained by BLBP and GFAP (scale bar, 50 μm). (B) Quantification of BLBP+ cell number in the CTX of Ctrl and FASD mice (*n* = 5/group). (C) Co‐staining of BLBP and Pax6 in the CTX of Ctrl and FASD mice at P21 (scale bar, 30 μm). (D) Quantification of BLBP+/Pax6+ cell number in the CTX of Ctrl and FASD mice at P21 (*n* = 5/group). (E) Western blotting showing BLBP, Pax6, and GFAP expression in the CTX of Ctrl and FASD mice at P21. (F) Quantification of BLBP, Pax6, and GFAP expression in the CTX of Ctrl and FASD mice (*n* = 4/group). Unpaired Student's *t*‐tests: (B, D, F). *p < 0.05, **p < 0.01, ***p < 0.001.

Previous studies have demonstrated that PAE induces the disruption of RGC development [24]. RGC is one of the main sources of OPC generation during embryonic and early postnatal stages. We assumed that the loss of OPCs in the FASD mice may be attributed to the failure of RGCs to transform into OPCs. BLBP staining showed an increased RGC number in the CTX of both male and female FASD mice at P21 (Figure 5A,B), while BLBP/Pax6 double staining revealed the elevated RGC number in the CTX (Figure 5C,D) and HIP (Figure S4C,D) of both male and female FASD mice at P21. Western blotting also showed aggregated BLBP and Pax6 expression in both male and female FASD mice (Figure 5E,F), indicating the disruption of RGCs to transform into distinct types of neural cells. On the other hand, it has been reported that PAE promoted the transformation of RGCs into astrocytes [25, 26], which may also contribute to the loss of OPCs in FASD mice. Interestingly, we found accumulated BLBP+/GFAP + double‐labeled reactive astrocytes in the CTX of male FASD mice, but not in the female (Figure 5A,B). The number and morphology of GFAP‐labeled reactive astrocytes were also found to be more severe in the HIP of male FASD mice compared to the female (Figure S4A,B), which is consistent with the variation of GFAP protein level being much higher in the CTX of male FASD mice than in females (Figure 5E,F). Finally, we checked the ability of RGCs to transform into OPCs in the FASD mice [30]. Pax6‐labeled PDGFRα+ OPCs were decreased in the CTX of both male and female mice (Figure S4E,F), demonstrating the failure of RGCs to differentiate into OPCs. Taken together, the sustained hypomyelination in the male FASD mice may be attributed to the combined effect of RGC dysfunction and severe astrocyte overactivation. This may partly explain the gender‐specific effect of FASD on CNS myelination.

### 3.5 Pro‐Myelination Drug Partially Rescues Hypomyelination and Hyperactivity‐Related Behaviors in the Male FASD Mice

At last, we wonder whether myelin restoration may participate in the regulation of behavioral abnormalities in the FASD mice. Clemastine fumarate (CF), a widely used FDA‐approved pro‐myelination drug that has been proven to be effective in promoting remyelination in myelin defect diseases and models [31, 32], was applied to the male FASD mice to detect its possible advantages in myelination and behaviors.

**FIGURE 6:**
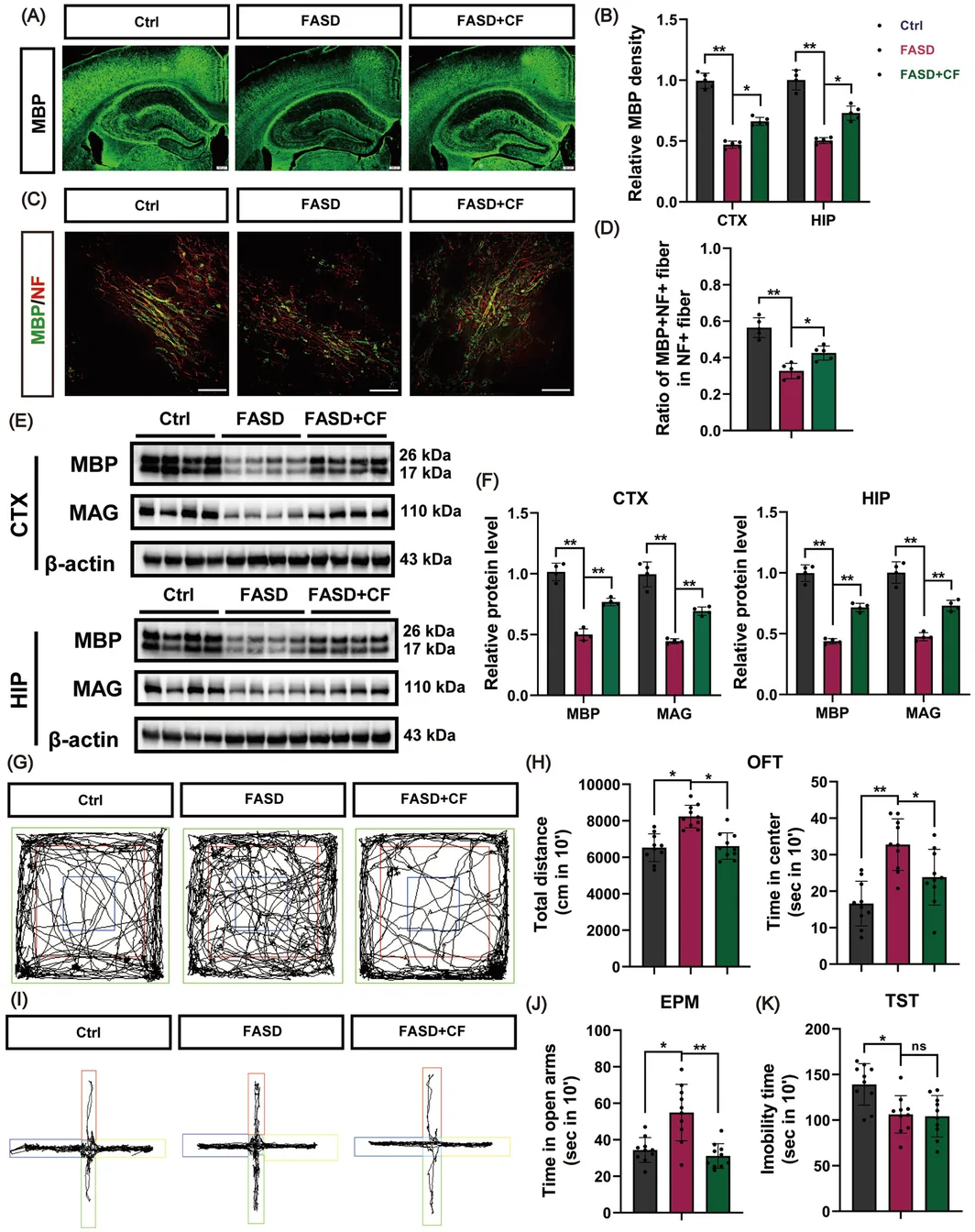
CF restores myelination and partially alleviates behavior abnormality in male FASD mice. (A) MBP staining showing myelin tracts in male Ctrl, FASD, and FASD+CF mice (scale bar, 200 μm). (B) Quantification of MBP density in male Ctrl, FASD, and FASD+CF mice (*n* = 5/group). (C) Co‐staining of MBP/NF showing ultrastructure of myelin in the CTX of male Ctrl, FASD, and FASD+CF mice (scale bar, 5 μm). (D) Quantification of the ratio of MBP+/NF+ fibers in NF+ fibers in the CTX of male Ctrl, FASD, and FASD+CF mice (*n* = 5/group). (E) Western blotting showing MBP and MAG expression in the CTX/HIP of male Ctrl, FASD, and FASD+CF mice. (F) Quantification of MBP and MAG expression in the CTX/HIP of male Ctrl, FASD, and FASD+CF mice (*n* = 4/group). (G) Diagram of the OFT for male Ctrl, FASD, and FASD+CF mice. (H) Total distance traveled and time spent in the center of the OFT of male Ctrl, FASD, and FASD+CF mice (*n* = 10/group). (I) Diagram of the EPM for male Ctrl, FASD, and FASD+CF mice. (J) Time spent in the open arms of the EPM of male Ctrl, FASD, and FASD+CF mice (*n* = 10/group). (K) Immobility time in the TST of male Ctrl, FASD, and FASD+CF mice (*n* = 10/group). One‐way ANOVA: (B, D, F, H, J, K). *p < 0.05, **p < 0.01.

MBP staining showed increased myelin density and expanded myelinated area in the CTX and HIP of CF‐treated male FASD mice (FASD+CF) compared with the FASD mice (Figure 6A,B). MBP/NF double staining revealed more myelinated axons and contact myelin sheath in the CTX of male FASD mice after CF treatment (Figure 6C,D). Western blotting showed that CF treatment also increased MBP and MAG expression in the CTX and HIP of male FASD mice (Figure 6E,F). These data demonstrated that CF treatment enhanced myelination in the male FASD mice.

Then we found that CF treatment partially alleviated the hyperactivity‐related behaviors of the male FASD mice compared with the saline‐treated FASD mice as follows: Decreased total travel and time spent in the center in the OFT (Figure 6G,H); less time spent in the open arms in the EPM (Figure 6I,J) but comparable immobility time in the TST (Figure 6K).

Additionally, CF was reported to enhance myelination via the coordination of astrocyte/microglia activation and neuroinflammation [33, 34, 35], we check whether the RGC‐mediated astrocyte overactivation can be corrected by CF in the male FASD mice. We found that the number of GFAP‐labeled reactive astrocytes was reduced after CF treatment in the CTX of male FASD mice (Figure S5A,B), and the morphology also showed less astrocyte reactivation after CF treatment (Figure S5A). Accordingly, GFAP protein level was significantly decreased after CF treatment in the CTX of male FASD mice (Figure S5C,D). These data demonstrated that CF treatment alleviated astrocyte overactivation in the male FASD mice. However, comparable BLBP and Pax6 expression were detected in the CTX of CF‐treated male FASD mice (Figure S5C,D), indicating that CF treatment may only ameliorate astrocyte overactivation, but not the process of RGCs transforming into reactive astrocytes.

Finally, we further checked the effects of CF on neuronal function in the male FASD mice. Western blotting showed that CF treatment increased SYP, but not PSD95 expression in the CTX of the male FASD mice (Figure S5E,F). Moreover, an ADHD‐related presynaptic protein, PTCHD1 [36, 37], whose expression was also elevated after CF treatment (Figure S5E,F). c‐Fos staining revealed blunted neural activity in the CTX of the male FASD mice, and CF treatment effectively aggregated c‐Fos expression in the FASD CTX (Figure S5G,H). Our data indicated that CF‐mediated remyelination may restored cortical neuron activity via a presynaptic manner.

## 4 Discussion

In the present study, we demonstrated impaired myelination and OL development in the male FASD mice, which is associated with hyperactivity‐related behaviors. However, the female FASD mice only exhibited delayed myelin formation, indicating a gender‐specific manner of myelination in FASD. Furthermore, decreased OPC proliferation was observed in both the male and female FASD mice, while only the male FASD mice exhibited that OPCs failed to differentiate into myelinated OLs. This may partly explain the sex‐dependent effect of FASD on myelination. Additionally, we demonstrate that FASD exerts an impact on the quantity and differentiation of RGCs into OPCs, consequently disrupting OL development and myelination. Finally, an FDA‐approved pro‐myelination drug, CF treatment in male FASD mice, restored myelination and consequently partially alleviated hyperactivity‐related behaviors. Together, our data reveal the deficiency of myelination and OL development in the CNS of the FASD mouse model and the possible underlying mechanism, which suggests a potential therapy target for FASD.

To characterize the FASD mice, we set up OFT, EPM, and TST to distinguish the behavioral features of the FASD offspring. We found that the female offspring showed normal behaviors in these three tests. However, the male offspring exhibited hyperactivity and increased exploratory behaviors, which were indicated by increased travel distance in the OFT and more time spent in the open arms of the EPM (Figure 1E–J). These findings are consistent with the ADHD‐like behaviors observed in the transgenic mouse models of dopamine transporter (DAT) deficiency syndrome [38, 39]. In addition, FASD is commonly characterized by hyperactivity, and it is reported that several individuals diagnosed as idiopathic attention‐deficit hyperactivity disorder (ADHD) due to PAE [14]. Clinical studies demonstrated that male FASD patients had higher rates of ADHD, whereas females experienced higher rates of depressive or mood disorders [40], which is consistent with hyperactivity‐like behaviors observed in the male FASD mice. However, we found less immobility of the male FASD offspring in the TST, which was reported inconsistencies during different ADHD models, in which hyperactivity is shown in all models [41, 42, 43]. These findings suggested partial behavioral consistency between ADHD and our FASD model. Moreover, alcohol exposure has been shown to be involved in cognitive deficiency, including learning and memory [44, 45, 46], indicating that more behavioral tests, such as the Morris water maze test (MWM), novel object recognition test (NOR), and Y‐maze test, should be helpful to specify the characterization of the male FASD mice. Additionally, a greater vulnerability of male offspring to the adverse effects of PAE has been reported [47]. Together with our data, these findings suggested the possible distinct neuropathology in male and female FASD mice.

Neuroimaging studies revealed abnormal structure of white matter in FASD patients or animal models [13, 14, 15, 16], indicating possible alteration of OLs or myelination in FASD. Indeed, our data showed a significant myelination defect during the postnatal developmental stage (from P14 to P90) in the male FASD mice. However, the female FASD mice only exhibited delayed myelin formation, in which myelin deficiency was observed at P14 and gradually restored at P21 and P90 (Figure 2). These results suggested a gender‐specific role of myelination in FASD. Accordingly, a diffusion tensor imaging (DTI) study revealed that boys with FASD exhibited higher cerebral fractional anisotropy (FA) than girls, which indicated more white matter damage in male FASD [48]. Consequently, this discrepancy led to reduced plasticity in the brain structural networks of adult males.

We next analyzed the possible mechanism of myelination deficiency in FASD. An accumulation of RGCs and BLBP expression were observed in the FASD mice, indicating that RGCs failed to transform into OPCs during postnatal development. Decreased OPC number was observed in both male and female FASD mice (Figure 3A–F), suggesting a common inhibition of OPC generation in both genders of FASD mice. Moreover, the EdU assay showed that OPC proliferation was only inhibited in the male FASD offspring at P21(Figure S3), which is consistent with the restoration of OL lineage cells and myelination in the female FASD mice after P21. These findings were consistent with the transcriptomics study in a postnatal mouse model of FASD, which showed decreased expression of the molecular markers of OL lineage cells, including OPCs and mature OLs [49]. However, the number of mature OLs was decreased in the male FASD mice, but not in the female (Figure 2G–L), indicating that only males with FASD exhibited a deficiency of OPC differentiation into myelinated OLs. This may partly result in the sex differences in myelination in FASD mice. Furthermore, accumulated BLBP+/GFAP+ labeled astrocytes and decreased Pax6+/PDGFRα+ labeled OPCs were observed in the CTX of male FASD mice, suggesting that RGCs was promoted to differentiate into astrocytes, but not into OPCs. Meanwhile, more severe overactivation of astrocytes was observed in the male FASD mice rather than the female. Astrocyte dysfunction was shown to disrupt myelination and OL function in multiple brain disorders [50, 51]. More accumulated GFAP+ labeled reactive astrocytes observed in the male FASD mice may have contributed to the sustained hypomyelination of the male with FASD. However, the precise role of RGCs in myelination and OL development needs to be clarified. For further research, transgenic tools to manipulate RGCs or OLs will be useful to clarify the exact roles of FASD in myelination.

Besides the effect of RGC dysfunction on myelination, NMDARs may also participate in the sex‐related myelination defect in the FASD mice. NMDARs expressed in the OPCs and OLs were shown to play critical roles in the developmental processes of oligodendrocytes and myelination [52, 53]. On the other hand, the sex‐specific effect of PAE on NMDAR function was reported [54, 55], which indicated the involvement of NMDARs in myelination control in the FASD mice in a gender‐dependent manner. In this case, OL lineage‐specific deletion of NMDARs is a useful tool to reveal the possible gender‐specific roles of NMDARs in myelination in the FASD mice.

At last, we explored the possible effects of the pro‐myelination approach on the myelin and behavior defects in the FASD mice. Pro‐myelinating drugs, such as CF, were widely used in multiple myelination defect‐related brain disease animal models and showed alleviation of symptoms [31, 32, 33, 34, 35]. In our study, we found that CF indeed restored myelination and partially alleviated hyperactivity‐related behaviors of the male FASD mice. Furthermore, CF treatment ameliorated astrocyte overactivation, which may also contribute to myelin maintenance and behavior alleviation in male FASD mice [33, 34]. Neuron activity monitored by c‐Fos also proved the advantages of CF treatment in male FASD mice. However, only the expression of presynaptic proteins, SYP and PTCHD1, was restored after CF treatment, which is consistent with the reconstruction of myelin and axon connection in the CF‐treated male FASD mice. PSD95 expression was not affected by CF, which indicated the distinct roles of PLAE in neuronal function besides myelination. Taken together, our data suggest pro‐myelinating drugs as a potential therapeutic approach for FASD.

Our study also has some limitations. We did not explore the exact role of RGC/astrocyte for myelination and OL development in the FASD mice. For further research, transgenic tools to manipulate RGCs or astrocytes during postnatal CNS development will be useful to clarify the exact roles of FASD in myelination.

## Author Contributions

All authors contributed to the study conception and design. Material preparation, data collection, and analysis were performed by Liang Zhou, Yi Zhang, Xiaoling Liang, and Rulan Yi. Zhidan Ke helped to generate the FASD model. The manuscript was written by Liang Zhou and Yi Zhang, and all authors commented on previous versions of the manuscript. All authors read and approved the final manuscript.

## Funding

This work was supported by the National Natural Science Foundation of China (82460242), the Scientific Project of Guizhou Province, (Qian Comprehensive Basic Science ZD[2026]160), and the project of Key Laboratory of Anesthesia and Organ Protection, Zunyi Medical University (3000311‐0008).

## Ethics Statement

All animal procedures complied with the ARRIVE guidelines and were conducted in accordance with the National Institutes of Health guide for the care and use of Laboratory animals (NIH Publications No. 8023, revised 1978) and the guidelines set forth by Zunyi Medical University (License Key. SYXK(Qian) 2021–0004).

## Conflicts of Interest

The authors declare no conflicts of interest.

## Supporting information


**Figure S1:** Quantification of MBP density in male and female FASD mice. (A) Quantification of MBP density in Ctrl and FASD mice at P14 (*n* = 5/group). (B) Quantification of MBP density in Ctrl and FASD mice at P21 (*n* = 5/group). (C) Quantification of MBP density in Ctrl and FASD mice at P90 (*n* = 5/group). (D) Co‐staining of MBP/NF showing ultrastructure of myelin in the CTX of Ctrl and FASD mice at P21 (scale bar, 5 μm). (E) Quantification of the ratio of MBP+/NF+ fibers in NF+ fibers in the CTX of Ctrl and FASD mice at P21 (*n* = 5/group). Unpaired Student's *t*‐tests: (A–C, E). **p < 0.01.
**FIGURE S2:** Decreased OPC number in both male and female FASD mice at P14. (A) Co‐staining of Olig2/CC‐1 showing total OLs (Olig2+) and mature (CC‐1+/Olig2+) OLs in the CTX of male Ctrl and FASD mice at P14 (scale bar, 50 μm). (B) Quantification of Olig2+ and CC‐1+/Olig2+ cell numbers in the CTX of male Ctrl and FASD mice at P14 (*n* = 5/group). (C) PDGFRα staining showing OPCs in the CTX of male Ctrl and FASD mice at P14 (scale bar, 50 μm). (D) Quantification of PDGFRα+ cell number in the CTX of male Ctrl and FASD mice at P14 (*n* = 5/group). (E) Co‐staining of Olig2/CC‐1 showing total OLs (Olig2+) and mature (CC‐1+/Olig2+) OLs in the CTX of female Ctrl and FASD mice at P14 (scale bar, 50 μm). (F) Quantification of Olig2+ and CC‐1+/Olig2+ cell numbers in the CTX of female Ctrl and FASD mice at P14 (*n* = 5/group). (G) PDGFRα staining showing OPCs in the CTX of female Ctrl and FASD mice at P14 (scale bar, 50 μm). (H) Quantification of PDGFRα+ cell number in the CTX of female Ctrl and FASD mice at P14 (*n* = 5/group). Unpaired Student's *t*‐tests: (B, D, F, H). *p < 0.05, **p < 0.01.
**FIGURE S3:** OPC proliferation is impaired in both male and female FASD mice at P14. (A) Co‐staining of PDGFRα/Edu showing the proliferating OPCs in the CTX of male Ctrl and FASD mice at P21 (scale bar, 50 μm). (B) Quantification of PDGFRα+/Edu + cell number in the CTX of male Ctrl and FASD mice at P21 (*n* = 5/group). (C) Co‐staining of PDGFRα/Edu showing the proliferating OPCs in the CTX of female Ctrl and FASD mice at P21 (scale bar, 50 μm). (D) Quantification of PDGFRα+/Edu + cell number in the CTX of female Ctrl and FASD mice at P21 (*n* = 5/group). (E) Co‐staining of Olig2/MBP showing OPC differentiation under alcohol treatment of the OPC culture (scale bar, 10 μm). (F) Quantification of differentiated OPC ratio in the OPC culture after alcohol treatment (*n* = 5/group). Unpaired Student's *t*‐tests: (B, D). **p < 0.01.
**FIGURE S4:** RGC failed to differentiate into OPC in FASD mice. (A) GFAP staining in the HIP of Ctrl and FASD mice at P21 (scale bar, 50 μm). (B) Quantification of GFAP+ astrocyte number in the HIP of Ctrl and FASD mice at P21 (*n* = 5/group). (C) Co‐staining of BLBP and Pax6 in the HIP of Ctrl and FASD mice at P21 (scale bar, 30 μm). (D) Quantification of BLBP+/Pax6+ cell number in the HIP of Ctrl and FASD mice at P21 (*n* = 5/group). (E) Co‐staining of PDGFRα and Pax6 in the CTX of Ctrl and FASD mice at P21 (scale bar, 30 μm). (F) Quantification of PDGFRα+/Pax6+ cell number in the CTX of Ctrl and FASD mice at P21 (*n* = 5/group). Unpaired Student's *t*‐tests: (B, D, F). *p < 0.05, **p < 0.01.
**FIGURE S5:** CF partially restored astrocyte and neuron function in the CTX of male FASD mice. (A) GFAP staining in the CTX of male Ctrl, FASD, and FASD+CF mice. Higher magnifications show the morphology of astrocytes in the CTX of the three groups (scale bar, 50 μm). (B) Quantification of GFAP+ astrocyte number in the CTX of male Ctrl, FASD, and FASD+CF mice (*n* = 5/group). (C) Western blotting showing GFAP, BLBP, and Pax6 expression in the CTX of male Ctrl, FASD, and FASD+CF mice. (D) Quantification of GFAP, BLBP, and Pax6 expression in the CTX of male Ctrl, FASD, and FASD+CF mice (*n* = 4/group). (E) Western blotting showing PSD95, SYP, and PTCHD1 expression in the CTX of male Ctrl, FASD, and FASD+CF mice. (F) Quantification of PSD95, SYP, and PTCHD1 expression in the CTX of male Ctrl, FASD, and FASD+CF mice (*n* = 4/group). (G) c‐Fos staining in the CTX of male Ctrl, FASD, and FASD+CF mice (scale bar, 50 μm). (H) Quantification of c‐Fos + astrocyte number in the CTX of male Ctrl, FASD, and FASD+CF mice (*n* = 5/group). One‐way ANOVA: (B, D, F, H). *p < 0.05, **p < 0.01.

## Data Availability

The data that support the findings of this study are available from the corresponding author upon reasonable request.
