# Supplementary material for: Developmental Deficiency of Oligodendrocytes and Myelination in the Central Nervous System of the Fetal Alcohol Syndrome Mouse Model
Source: CNS Neurosci Ther. 2026 Sep 11;32(9):e71157. doi: 10.1002/cns.71157 (PMC13570189; doi:10.1002/cns.71157)
Supplement: Supplementary file 1 [file CNS-32-e71157-s001.docx]

**FIGURE S1. Quantification of MBP density in male and female FASD mice.** (A) Quantification of MBP density in Ctrl and FASD mice at P14 (n=5/group). (B) Quantification of MBP density in Ctrl and FASD mice at P21 (n=5/group). (C) Quantification of MBP density in Ctrl and FASD mice at P90 (n=5/group). (D) Co-staining of MBP/NF showing ultra structure of myelin in the CTX of Ctrl and FASD mice at P21 (scale bar, 5 μm). (E) Quantification of the ratio of MBP+/NF+ fiber in NF+ fiber in the CTX of Ctrl and FASD mice at P21 (n=5/group). Unpaired Student’s t-tests: (A-C, E). ***p*＜0.01.

**FIGURE S2. Decreased OPC number in both male and female FASD mice at P14.** (A) Co-staining of Olig2/CC-1 showing total OLs (Olig2+) and mature (CC-1+ /Olig2+) OLs in the CTX of male Ctrl and FASD mice at P14 (scale bar, 50 μm). (B) Quantification of Olig2+ and CC-1+/Olig2+ cell number in the CTX of male Ctrl and FASD mice at P14 (n=5/group). (C) PDGFRα staining showing OPC in the CTX of male Ctrl and FASD mice at P14 (scale bar, 50 μm). (D) Quantification of PDGFRα+ cell number in the CTX of male Ctrl and FASD mice at P14 (n=5/group). (E) Co-staining of Olig2/CC-1 showing total OLs (Olig2+) and mature (CC-1+ /Olig2+) OLs in the CTX of female Ctrl and FASD mice at P14 (scale bar, 50 μm). (F) Quantification of Olig2+ and CC-1+/Olig2+ cell number in the CTX of female Ctrl and FASD mice at P14 (n=5/group). (G) PDGFRα staining showing OPC in the CTX of female Ctrl and FASD mice at P14 (scale bar, 50 μm). (H) Quantification of PDGFRα+ cell number in the CTX of female Ctrl and FASD mice at P14 (n=5/group). Unpaired Student’s t-tests: (B, D, F, H). **p*＜0.05, ***p*＜0.01.

**FIGURE S3. OPC proliferation is impaired in both male and female FASD mice at P14.** (A) Co-staining of PDGFRα/Edu showing the proliferating OPCs in the CTX of male Ctrl and FASD mice at P21 (scale bar, 50 μm). (B) Quantification of PDGFRα+/Edu+ cell number in the CTX of male Ctrl and FASD mice at P21 (n=5/group). (C) Co-staining of PDGFRα/Edu showing the proliferating OPCs in the CTX of female Ctrl and FASD mice at P21 (scale bar, 50 μm). (D) Quantification of PDGFRα+/Edu+ cell number in the CTX of female Ctrl and FASD mice at P21 (n=5/group). (E) Co-staining of Olig2/MBP showing OPC differentiation under alcohol treatment of the OPC culture (scale bar, 10 μm). (F) Quantification of differentiated OPC ratio in the OPC culture after alcohol treatment (n=5/group). Unpaired Student’s t-tests: (B, D). ***p*< 0.01.

**FIGURE S4. RGC failed to differentiate into OPC in FASD mice.** (A) GFAP staining in the HIP of Ctrl and FASD mice at P21(scale bar, 50 μm). (B) Quantification of GFAP+ astrocyte number in the HIP of Ctrl and FASD mice at P21 (n=5/group). (C) Co-staining of BLBP and Pax6 in the HIP of Ctrl and FASD mice at P21 (scale bar, 30 μm). (D) Quantification of BLBP+/Pax6+ cell number in the HIP of Ctrl and FASD mice at P21 (n=5/group). (E) Co-staining of PDGFRα and Pax6 in the CTX of Ctrl and FASD mice at P21 (scale bar, 30 μm). (F) Quantification of PDGFRα+/Pax6+ cell number in the CTX of Ctrl and FASD mice at P21 (n=5/group). Unpaired Student’s t-tests: (B, D, F). **p*< 0.05, ***p*< 0.01.

**FIGURE S5. CF partially restored astrocyte and neuron function in the CTX of male FASD mice.** (A) GFAP staining in the CTX of male Ctrl, FASD and FASD+CF mice. Higher magnifications show the morphology of astrocytes in the CTX of the three groups (scale bar, 50 μm). (B) Quantification of GFAP+ astrocyte number in the CTX of male Ctrl, FASD and FASD+CF mice (n=5/group). (C) Western blotting showing GFAP, BLBP and Pax6 expression in the CTX of male Ctrl, FASD and FASD+CF mice. (D) Quantification of GFAP, BLBP and Pax6 expression in the CTX of male Ctrl, FASD and FASD+CF mice (n=4/group). (E) Western blotting showing PSD95, SYP and PTCHD1 expression in the CTX of male Ctrl, FASD and FASD+CF mice. (F) Quantification of PSD95, SYP and PTCHD1 expression in the CTX of male Ctrl, FASD and FASD+CF mice (n=4/group). (G) c-Fos staining in the CTX of male Ctrl, FASD and FASD+CF mice (scale bar, 50 μm). (H) Quantification of c-Fos+ astrocyte number in the CTX of male Ctrl, FASD and FASD+CF mice (n=5/group). One-way ANOVA: (B, D, F, H). **p*< 0.05, ***p*< 0.01.
